# A monoclinic polymorph of [(*Z*)-*N*-(3-chloro­phen­yl)-*O*-methyl­thio­carbamato-κ*S*](tri­phenyl­phosphane-κ*P*)gold(I): crystal structure and Hirshfeld surface analysis

**DOI:** 10.1107/S2056989016010781

**Published:** 2016-07-07

**Authors:** Chien Ing Yeo, Sang Loon Tan, Edward R. T. Tiekink

**Affiliations:** a Research Centre for Crystalline Materials, Faculty of Science and Technology, Sunway University, 47500 Bandar Sunway, Selangor Darul Ehsan, Malaysia

**Keywords:** crystal structure, polymorph, gold, thio­carbamate, Hirshfeld surface analysis

## Abstract

The title compound, [Au(C_8_H_7_ClNOS)(C_18_H_15_P)], is a monoclinic (*P*2_1_/*n*, *Z*′ = 1; form β) polymorph of the previously reported triclinic form (*P*-1, *Z*′ = 1; form α) [Tadbuppa & Tiekink (2010 ▸). *Acta Cryst*. E**66**, m664]. The mol­ecular structures of both forms feature an almost linear gold(I) coordination geometry [P—Au—S = 175.62 (5)° in the title polymorph], being coordinated by thiol­ate S and phosphane P atoms, a *Z* conformation about the C=N bond and an intra­molecular Au⋯O contact. The major conformational difference relates to the relative orientations of the residues about the Au—S bond: the P—Au—S—C torsion angles are −8.4 (7) and 106.2 (7)° in forms α and β, respectively. The mol­ecular packing of form β features centrosymmetric aggregates sustained by aryl-C—H⋯O inter­actions, which are connected into a three-dimensional network by aryl-C—H⋯π contacts. The Hirshfeld analysis of forms α and β shows many similarities with the notable exception of the influence of C—H⋯O inter­actions in form β.

## Abstract 2

A linear geometry defined by a *P*,*S*-donor set is observed in the title polymorph; an intra­molecular Au⋯O inter­action is noted. The packing is consolidated by C—H⋯O and C—H⋯π inter­actions to generate a three-dimensional network.

## Chemical context   

Inter­est in the chemistry of phosphanegold(I) *N*-aryl-*O*-alkyl­thio­carbamates, *i.e*. compounds of general formula *R*_3_PAu[SC(O*R*′)=N*R*′′] (*R*, *R*′ = alkyl, aryl; *R*′′ = ar­yl) continues owing to their recently disclosed exciting biological activities. Thus, various tri­phenyl­phosphane derivatives display excellent cytotoxicity profiles against HT-29 colon cancer cells, a particularly virulent form of cancer, and mechanistic studies have shown these to induce both intrinsic and extrinsic pathways of cell death leading to apoptosis (Yeo, Ooi *et al.*, 2013 ▸; Ooi *et al.*, 2015 ▸). Further, species with *R*′′ = *p*-tolyl have proven to exhibit impressive *in vitro* potency against Gram-positive bacteria (Yeo, Sim *et al.*, 2013 ▸). It was during another synthesis of the title compound, (I), for further biological studies, that crystals of a new polymorph were isolated from its methanol solution. This is called form β to distinguish it from the earlier triclinic form, form α (Tadbuppa & Tiekink, 2010 ▸). Herein, the crystal and mol­ecular structures of form β of (I) are described along with a comparison with the parameters characterizing form α. Further, a Hirshfeld surface analysis of both polymorphic forms of (I) is presented.

## Structural commentary   

**Figure 1:**
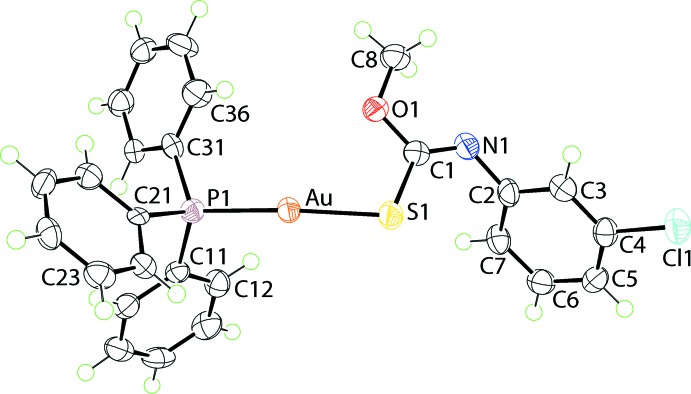
The mol­ecular structure of polymorphic form β of (I), showing the atom-labelling scheme and displacement ellipsoids at the 70% probability level.

The mol­ecular structure of the new monoclinic form of (I), form β, is shown in Fig. 1 ▸, and selected geometric parameters are collected in Table 1 ▸. The gold(I) atom is coordinated in an approximately linear configuration by phosphane-P and thiol­ate-S atoms. Confirmation of the ‘thiol­ate’ assignment is readily seen in the relatively long C1—S1 bond length and the significant π-character in the C1—N1 bond when the geometric parameters are compared with structures of related thio­carbamide mol­ecules (Ho *et al.*, 2005 ▸; Kuan *et al.*, 2007 ▸); the crystal structure of the thio­carbamide precursor in (I) is not available for comparison. As is invariably observed in this class of compound, the Au—S bond length is longer than the Au—P bond. The small deviation from ideal linearity for the P—Au—S bond is related to the close approach of the oxygen atom to the gold(I) atom, *i.e*. 3.052 (3) Å. The pattern of bond angles about the quaternary carbon atom, C1, follow the expected trends with the widest angle involving the sulfur and doubly bonded nitro­gen atom and with the narrowest angle involving the single-bonded atoms. The conformation about the formal C1=N1 bond, Table 1 ▸, is *Z*.
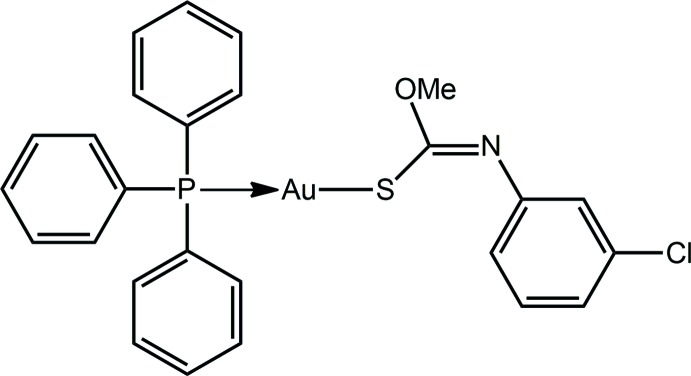


**Figure 2:**
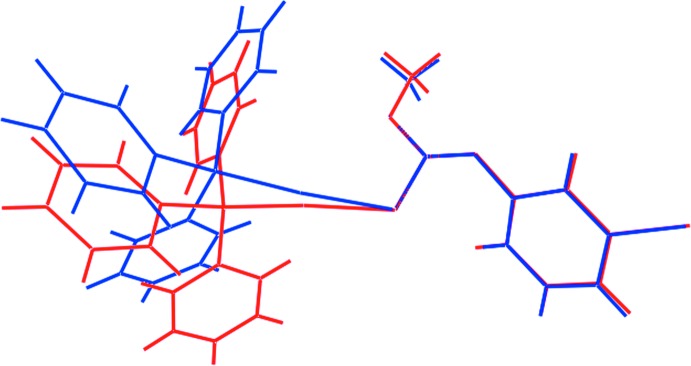
Overlay diagram of polymorphic forms α (blue image) and β (red) of the mol­ecular structures of (I). Mol­ecules have been overlapped so that the S1, O1 and N1 atoms are coincident.

Form β crystallizes in the monoclinic space group *P*2_1_/*n* with *Z*′ = 1. The earlier polymorph, by contrast, crystallizes in triclinic space group *P* 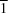, also with *Z*′ = 1. A comparison of the key geometric parameters is given in Table 1 ▸. From these data, it is clear that there is experimental inequivalence in the bond lengths involving the gold(I) atoms, with the Au—S and Au—P bond lengths in form β being marginally longer. The intra­molecular Au⋯O separation in form β is also longer than the comparable separation in form α, and this is correlated with a smaller deviation from a linear geometry about the gold(I) atom in β. By contrast, the bond angles are, by and large, equivalent within experimental error. A significant conformational difference is evident in the mol­ecular structures of forms α and β of (I). As seen from the overlay diagram shown in Fig. 2 ▸, this difference occurs as a result of a twist about the Au—S bond as seen in the values of the P1—Au—S1—C1 torsion angles of −8.4 (7) and 106.2 (7)° in forms α and β, respectively.

## Supra­molecular features   

**Figure 3:**
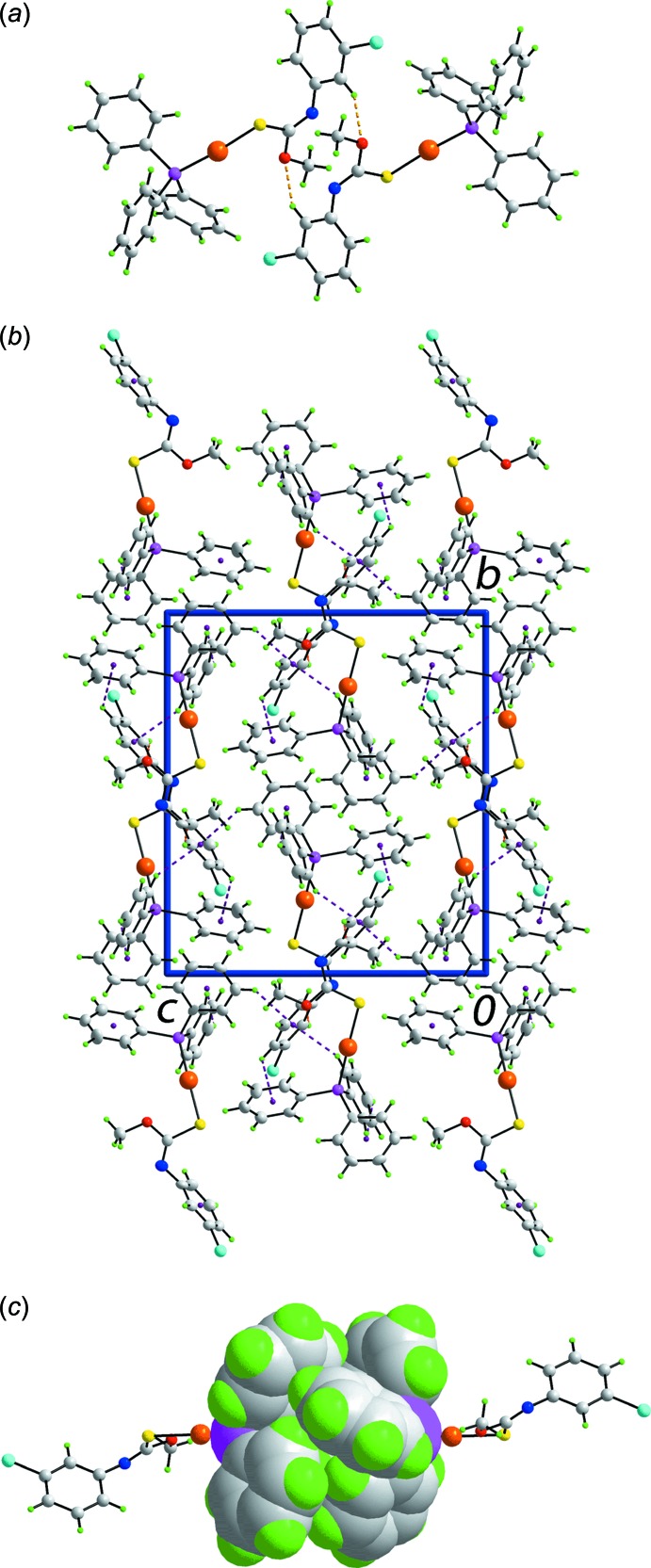
Mol­ecular packing in form β of (I): (*a*) view of the supra­molecular dimer sustained by C—H⋯O contacts, shown as orange dashed lines, (*b*) view of the unit-cell contents shown in projection down the *a* axis, highlighting the C—H⋯π inter­actions as purple dashed lines, (*c*) image of the sixfold phenyl (6PE) between centrosymmetrically related Ph_3_P ligands, highlighted in space-filling mode.

Supra­molecular dimers feature in the mol­ecular packing of form β of (I), which are sustained by N-aryl-C—H⋯O(meth­oxy) inter­actions, Fig. 3 ▸ *a* and Table 2 ▸. The dimers are connected into a three-dimensional architecture by a network of C—H⋯π inter­actions, Fig. 3 ▸ *b* and Table 2 ▸. Within this arrangement, centrosymmetrically related Ph_3_P ligands align to form a so-called six-fold phenyl embrace (6PE) (Dance & Scudder, 1995 ▸) featuring edge-to-face phenyl-C—H⋯π(phen­yl) inter­actions, Fig. 3 ▸ *c*. While the inter­actions are too long to be considered as significant in terms of the criteria in *PLATON* (Spek, 2009 ▸), there are a number of such inter­actions, *i.e*. 2 × [3.22, 3.26 and 3.29 Å], that serve to reinforce the 6PE embrace with one pair of rings accepting two inter­actions each. In form α of (I), the most prominent feature of the mol­ecular packing is the formation of supra­molecular chains mediated by C—H⋯π inter­actions (Tadbuppa & Tiekink, 2010 ▸). Further analysis of the mol­ecular packing in polymorphic (I) is given in the following Section.

## Analysis of the Hirshfeld surfaces   

The non-covalent inter­actions present in the pair of polymorphs of (I), *i.e*. forms α and β, were studied through Hirshfeld surface analysis by mapping on the normalized contact distance (*d*_norm_) upon computation of the inner (*d*_i_) and outer (*d*_e_) distances of the Hirshfeld surface to the nearest nucleus (Spackman & Jayatilaka, 2009 ▸; McKinnon *et al.*, 2007 ▸). All computation as well as generation of two-dimensional fingerprint plots were performed using *Crystal Explorer 3.1* (Wolff *et al.*, 2012 ▸). Distances involving hydrogen atoms were normalized by default to the standard neutron-diffraction bond lengths.

**Figure 4:**
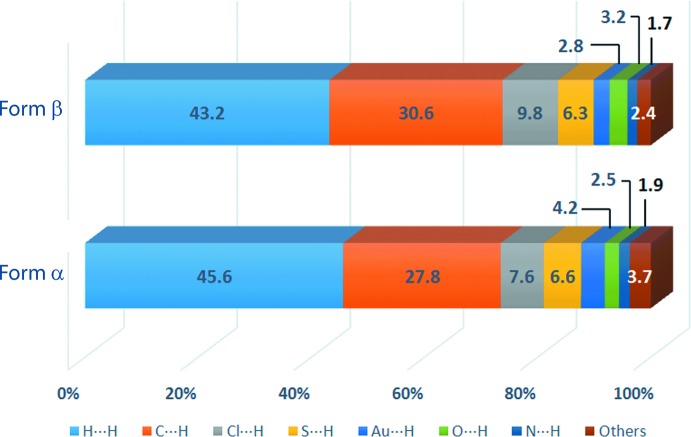
Percentage contribution of different close contacts to the Hirshfeld surface of forms α and β of (I).

**Figure 5:**
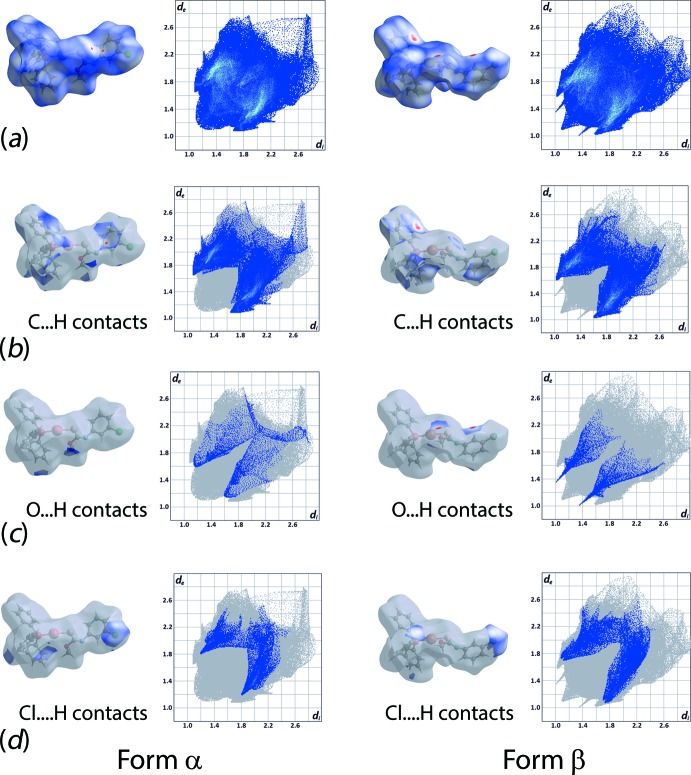
Comparison of the (*a*) complete Hirshfeld surface and full fingerprint plots between form α and form β polymorphs (top row) and the corresponding *d*_norm_ surfaces and two-dimensional plots associated with (*b*) C⋯H/H⋯C, (*c*) O⋯H/H⋯O and (*d*) Cl⋯H/H⋯Cl contacts.

As evident from Fig. 4 ▸ and Table 3 ▸, forms α and β of (I) exhibit relatively similar percentage contributions of the indicated inter­molecular inter­actions to their Hirshfeld surfaces. However, the specific contributions to their inter­action profiles are distinct as evidenced from the overall and decomposed two-dimensional fingerprint plots shown in Fig. 5 ▸. As mentioned above in *Supra­molecular features*, C—H⋯π inter­actions feature in both structures. To a first approximation the decomposed fingerprint plots look similar, as seen from Fig. 5 ▸ *b*. However, relatively shorter contacts are found in form β *cf*. form α, *i.e*. 2.62 *vs* 2.68 Å. The clear distinction between the two forms is readily noted from the decomposed fingerprint plots for the O⋯H/H⋯O contacts with very distinct spikes evident for form β, Fig. 5 ▸ *c*, correlating with the C—H⋯O inter­actions leading to dimer formation. While beyond the sum of their respective van der Waals radii (Spek, 2009 ▸), Cl⋯H/H⋯Cl inter­actions make contributions to the Hirshfeld surfaces of both forms α and β, with the contacts, again, being shorter in form β, *i.e*. 2.76 *vs* 3.00 Å, leading to more the distinct forceps in Fig. 5 ▸ *d*.

In general, the observation of generally shorter contacts in form β may indicate greater crystal-packing efficiency (Lloyd *et al.*, 2005 ▸). Table 4 ▸ collates various mol­ecular/crystal structure descriptors for the polymorphic forms. Immediately evident is that the calculated unit-cell densities are identical but the crystal-packing efficiency (KPI; Spek, 2009 ▸) for form β is marginally greater. Computation on the area-to-volume ratio between forms α and β revealed very little difference as did the globularity (*G*) and asphericity (Ω) indices. All these indicators suggest that the polymorphs arise as a result of a simple inter­play between mol­ecular conformation and crystal-packing effects.

## Database survey   

The most closely related structure to (I) in the crystallographic literature (Groom *et al.*, 2016 ▸), is the *R*′ = OEt analogue, *i.e*. (II), (Tadbuppa & Tiekink, 2009 ▸). Key geometric parameters for this structure are also included in Table 1 ▸. Non-systematic variations in parameters are noted, *e.g*. the Au—S bond length in (II) is inter­mediate between those found in the polymorphic forms of (I), and the Au—P bond length is the longest of the three structures. However, differences are small and probably can be ascribed to the influences of crystal-packing effects.

As indicated in the *Chemical context*, biological considerations motivate ongoing investigations into the chemistry of phosphanegold(I) *N*-aryl-*O*-alkyl­thio­carbamates. This notwithstanding, the relative ease of growing crystals have prompted several crystal engineering studies. Thus, correlations between Au⋯Au (aurophilic) and solid-state luminescence responses have been made for the series of compounds, *R*_3_PAu[SC(OMe)=NC_6_H_4_NO_2_-*p*] (*R* = Et, Cy and Ph), and bidentate phosphane analogues, Ph_2_P–(CH_2_)_*n*_–PPh_2_ for *n* = 1–4 and when the bridge is Fc (ferrocen­yl) (Ho *et al.*, 2006 ▸). In another study, the influence of *R* and *Y* substituents upon the mol­ecular packing of compounds of the general formula [(Ph_2_P(CH_2_)_4_PPh_2_){AuSC(O*R*′)=NC_6_H_4_ *Y*-*p*}_2_] for *R*′ = Me, Et or *i*Pr and *Y* = H, NO_2_ or Me was undertaken (Ho & Tiekink, 2007 ▸). Besides the anti­cipated linear P—Au—S configuration, a common feature of all the analysed structures until then was the presence of intra­molecular Au⋯O inter­actions, as illustrated in Fig. 1 ▸. This changed in another systematic study, this time of *R*_3_PAu[SC(OMe)=N*R*′′], for *R* = Ph, *o*-tol, *m*-tol or *p*-tol, and *R′*’ = Ph, *o*-tol, *m*-tol, *p*-tol or C_6_H_4_NO_2_-*p*, where it proved possible to induce a conformational change in the mol­ecule so that an intra­molecular Au⋯π inter­action formed rather than Au⋯O (Kuan *et al.*, 2008 ▸); Au⋯π inter­actions are well documented in the crystallographic literature (Tiekink & Zukerman-Schpector, 2009 ▸; Caracelli *et al.*, 2013 ▸). For example, having *R* = *R*′′ = *p*-tol simultaneously activated the gold atom, making it amenable to form an Au⋯π inter­action with the comparatively electron-rich aryl ring. Recently, bipodal forms of the thio­carbamide ligands were prepared and complexed with phosphanegold(I) species yielding binuclear mol­ecules also with intra­molecular Au⋯π inter­actions (Yeo *et al.*, 2015 ▸). Computational chemistry showed the Au⋯π inter­actions to be more favourable, by *ca* 12 kcal mol^−1^, than the putative Au⋯O inter­action (Yeo *et al.*, 2015 ▸).

Such inter­play between substituents in crystal engineering endeavours, along with the observation that biological activities are acutely sensitive to substitution patterns, ensures this area of research will continue to attract significant attention.

## Synthesis and crystallization   

All chemicals and solvents were used as purchased without purification. All reactions were carried out under ambient conditions. Melting points were determined on a Biobase auto melting point apparatus MP300. IR spectra were obtained on a Perkin Elmer Spectrum 400 FT Mid-IR/Far-IR spectrophotometer from 4000 to 400 cm^−1^; abbreviation: s, strong.

Preparation of (I): NaOH (Merck; 0.25 mmol, 0.01 g) in MeOH (Merck; 1 ml) was added to a suspension of Ph_3_PAuCl (0.25 mmol, 0.12 g) in MeOH (Merck; 15 ml), followed by addition of the thio­carbamide, MeOC(=S)N(H)C_6_H_4_Cl_3_ (0.25 mmol, 0.05 g), prepared following literature precedents (Ho *et al.*, 2005 ▸), in MeOH (15 ml). The resulting mixture was stirred for 2 h at 323 K. The solution mixture was left for slow evaporation at room temperature, yielding colourless prisms of the title compound after 3 weeks. Yield: 0.134 g (81%). M.p. 431–433 K. IR (cm^−1^): 1434 (*s*) ν(C=N), 1180 (*s*) ν(C—O), 1098 (*s*) ν(C—S).

## Refinement   

Crystal data, data collection and structure refinement details are summarized in Table 5 ▸. The carbon-bound H atoms were placed in calculated positions (C—H = 0.95–0.98 Å) and were included in the refinement in the riding-model approximation, with *U*_iso_(H) set to 1.2–1.5*U*_eq_(C). The maximum and minimum residual electron density peaks of 2.04 and 1.06 e Å^−3^, respectively, were located 1.01 and 0.77 Å from the Au atom.

## Supplementary Material

Crystal structure: contains datablock(s) I, global. DOI: 10.1107/S2056989016010781/hb7598sup1.cif

Structure factors: contains datablock(s) I. DOI: 10.1107/S2056989016010781/hb7598Isup2.hkl

CCDC reference: 1489737

Additional supporting information: crystallographic information; 3D view; checkCIF report

## Figures and Tables

**Table 1:** Geometric data (Å, °) for (I), forms α^*a*^ and β, and (II)^*b*^

Parameter	(I): form α	(I): form β	(II)
Au—S1	2.2902 (13)	2.3070 (14)	2.3041 (9)
Au—P1	2.2416 (11)	2.2535 (14)	2.2588 (8)
C1—S1	1.760 (5)	1.764 (5)	1.759 (4)
C1—O1	1.355 (6)	1.362 (6)	1.356 (4)
C1—N1	1.241 (6)	1.274 (6)	1.265 (4)
Au⋯O1	2.988 (3)	3.052 (3)	2.967 (3)
S1—Au—P1	174.61 (4)	175.62 (5)	175.86 (3)
Au—S1—C1	102.46 (16)	101.78 (18)	103.15 (12)
C1—O1—C8	116.8 (4)	115.4 (4)	117.8 (3)
C1—N1—C2	120.4 (4)	120.8 (5)	119.6 (3)
S1—C1—O1	113.0 (3)	112.6 (4)	111.9 (2)
S1—C1—N1	126.6 (4)	127.7 (4)	127.7 (3)
O1—C1—N1	120.4 (4)	119.7 (5)	120.3 (3)

Notes: (*a*) Tadbuppa & Tiekink (2010 ▸); (*b*) Tadbuppa & Tiekink (2009 ▸).

**Table 2:** Hydrogen-bond geometry (Å, °) Hydrogen-bond geometry (Å, °), form β, *Cg*1, *Cg*3 and *Cg*4 are the centroids of the C2–C7, C21–C26 and C31–C36 rings, respectively.

*D*—H⋯*A*	*D*—H	H⋯*A*	*D*⋯*A*	*D*—H⋯*A*
C3—H3⋯O1^i^	0.95	2.47	3.315 (7)	148
C5—H5⋯*Cg*4^ii^	0.95	2.85	3.492 (6)	126
C12—H12⋯*Cg*1^ii^	0.95	2.64	3.450 (6)	143
C14—H14⋯*Cg*3^iii^	0.95	2.80	3.570 (6)	139
C23—H23⋯*Cg*1^iv^	0.95	2.65	3.435 (6)	140

Symmetry codes: (i) 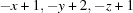; (ii) 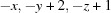; (iii) 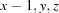; (iv) 
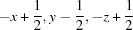
.

**Table 3:** Percentage contribution of the different inter­molecular contacts to the Hirshfeld surface in forms α and β of (I)

Contact	% Contribution form α	% Contribution form β
Au⋯Cl	0.2	0.6
Au⋯C	0.3	0.2
Au⋯H	4.2	2.8
Cl⋯C	2.7	0.3
Cl⋯H	7.6	9.8
Cl⋯S	0.0	0.2
S⋯C	0.1	0.0
S⋯H	6.6	6.3
O⋯H	2.5	3.2
N⋯H	1.9	1.7
N⋯C	0.0	0.3
C⋯C	0.4	0.8
C⋯H	27.8	30.6
H⋯H	45.6	43.2
Total	99.9	100

**Table 4:** Physiochemical properties for forms α and β of (I)

Parameter	Form α	Form β
Volume, *V* (Å^3^)	596.42	596.78
Surface area, *A* (Å^2^)	518.92	511.11
*A*:*V* (Å^−1^)	0.87	0.86
Globularity, *G*	0.660	0.671
Asphericity, Ω	0.165	0.172
Density (g cm^−1^)	1.805	1.805
Packing index (%)	66.9	67.4

**Table 5:** Experimental details

Crystal data	
Chemical formula	[Au(C_8_H_7_ClNOS)(C_18_H_15_P)]
*M*_r_	659.89
Crystal system, space group	Monoclinic, *P*2_1_/*n*
Temperature (K)	100
*a*, *b*, *c* (Å)	9.0078 (4), 17.4732 (7), 15.5641 (7)
β (°)	97.595 (4)
*V* (Å^3^)	2428.22 (18)
*Z*	4
Radiation type	Mo *K*α
μ (mm^−1^)	6.34
Crystal size (mm)	0.10 × 0.05 × 0.03

Data collection	
Diffractometer	Agilent SuperNova Dual Source diffractometer with an Atlas detector
Absorption correction	Multi-scan (*CrysAlis PRO*; Agilent, 2010 ▸)
*T*_min_, *T*_max_	0.570, 0.833
No. of measured, independent and observed [*I* > 2σ(*I*)] reflections	18211, 5613, 4470
*R*_int_	0.065
(sin θ/λ)_max_ (Å^−1^)	0.651

Refinement	
*R*[*F* ^2^ > 2σ(*F* ^2^)], *wR*(*F* ^2^), *S*	0.040, 0.090, 1.05
No. of reflections	5613
No. of parameters	290
H-atom treatment	H-atom parameters constrained
Δρ_max_, Δρ_min_ (e Å^−3^)	2.04, −1.06

Computer programs: *CrysAlis PRO* (Agilent, 2010 ▸), *SHELXS97* (Sheldrick, 2008 ▸), *SHELXL2014* (Sheldrick, 2015 ▸), *ORTEP-3 for Windows* (Farrugia, 2012 ▸), *QMol* (Gans & Shalloway, 2001 ▸), *DIAMOND* (Brandenburg, 2006 ▸) and *publCIF* (Westrip, 2010 ▸).

## supplementary crystallographic information

### Crystal data

**Table:**

[Au(C_8_H_7_ClNOS)(C_18_H_15_P)]	*F*(000) = 1280
*M**_r_* = 659.89	*D*_x_ = 1.805 Mg m^−^^3^
Monoclinic, *P*2_1_/*n*	Mo *K*α radiation, λ = 0.71073 Å
*a* = 9.0078 (4) Å	Cell parameters from 5344 reflections
*b* = 17.4732 (7) Å	θ = 2.3–27.5°
*c* = 15.5641 (7) Å	µ = 6.34 mm^−^^1^
β = 97.595 (4)°	*T* = 100 K
*V* = 2428.22 (18) Å^3^	Prism, colourless
*Z* = 4	0.10 × 0.05 × 0.03 mm

### Data collection

**Table:**

Agilent SuperNova Dual Source diffractometer with an Atlas detector	5613 independent reflections
Radiation source: SuperNova (Mo) X-ray Source	4470 reflections with *I* > 2σ(*I*)
Mirror monochromator	*R*_int_ = 0.065
Detector resolution: 10.4041 pixels mm^-1^	θ_max_ = 27.6°, θ_min_ = 2.3°
ω scan	*h* = −11→9
Absorption correction: multi-scan (CrysAlis PRO; Agilent, 2010)	*k* = −22→22
*T*_min_ = 0.570, *T*_max_ = 0.833	*l* = −17→20
18211 measured reflections	

### Refinement

**Table:**

Refinement on *F*^2^	0 restraints
Least-squares matrix: full	Hydrogen site location: inferred from neighbouring sites
*R*[*F*^2^ > 2σ(*F*^2^)] = 0.040	H-atom parameters constrained
*wR*(*F*^2^) = 0.090	*w* = 1/[σ^2^(*F*_o_^2^) + (0.0282*P*)^2^] where *P* = (*F*_o_^2^ + 2*F*_c_^2^)/3
*S* = 1.05	(Δ/σ)_max_ < 0.001
5613 reflections	Δρ_max_ = 2.04 e Å^−^^3^
290 parameters	Δρ_min_ = −1.06 e Å^−^^3^

### Special details

**Table:**

Geometry. All esds (except the esd in the dihedral angle between two l.s. planes) are estimated using the full covariance matrix. The cell esds are taken into account individually in the estimation of esds in distances, angles and torsion angles; correlations between esds in cell parameters are only used when they are defined by crystal symmetry. An approximate (isotropic) treatment of cell esds is used for estimating esds involving l.s. planes.

### Fractional atomic coordinates and isotropic or equivalent isotropic displacement parameters (Å^2^)

**Table:**

	*x*	*y*	*z*	*U*_iso_*/*U*_eq_	
Au	0.19155 (2)	0.79563 (2)	0.43242 (2)	0.02135 (8)	
Cl1	0.35621 (18)	1.26890 (8)	0.33228 (10)	0.0342 (4)	
P1	0.11209 (17)	0.67786 (8)	0.46382 (9)	0.0209 (3)	
S1	0.25415 (18)	0.91846 (8)	0.39637 (9)	0.0263 (3)	
O1	0.3390 (4)	0.9118 (2)	0.5632 (2)	0.0231 (8)	
N1	0.2776 (5)	1.0330 (2)	0.5172 (3)	0.0238 (10)	
C1	0.2894 (6)	0.9623 (3)	0.4991 (3)	0.0206 (11)	
C2	0.2162 (7)	1.0859 (3)	0.4530 (4)	0.0240 (12)	
C3	0.3063 (6)	1.1426 (3)	0.4247 (3)	0.0222 (12)	
H3	0.4107	1.1438	0.4442	0.027*	
C4	0.2408 (7)	1.1975 (3)	0.3675 (4)	0.0237 (12)	
C5	0.0901 (7)	1.1990 (3)	0.3373 (4)	0.0231 (12)	
H5	0.0489	1.2371	0.2975	0.028*	
C6	0.0000 (7)	1.1425 (3)	0.3670 (4)	0.0270 (13)	
H6	−0.1046	1.1423	0.3478	0.032*	
C7	0.0618 (6)	1.0863 (3)	0.4249 (4)	0.0262 (13)	
H7	−0.0007	1.0484	0.4452	0.031*	
C8	0.3796 (7)	0.9450 (3)	0.6480 (3)	0.0294 (13)	
H8A	0.4077	0.9042	0.6903	0.044*	
H8B	0.2941	0.9735	0.6647	0.044*	
H8C	0.4645	0.9798	0.6466	0.044*	
C11	−0.0850 (6)	0.6656 (3)	0.4249 (3)	0.0208 (12)	
C12	−0.1829 (7)	0.7251 (3)	0.4386 (4)	0.0258 (13)	
H12	−0.1448	0.7711	0.4657	0.031*	
C13	−0.3350 (7)	0.7173 (3)	0.4127 (4)	0.0316 (14)	
H13	−0.4014	0.7575	0.4228	0.038*	
C14	−0.3908 (7)	0.6504 (3)	0.3719 (4)	0.0301 (14)	
H14	−0.4953	0.6451	0.3544	0.036*	
C15	−0.2952 (7)	0.5923 (3)	0.3569 (4)	0.0289 (13)	
H15	−0.3340	0.5474	0.3277	0.035*	
C16	−0.1436 (6)	0.5982 (3)	0.3838 (3)	0.0252 (12)	
H16	−0.0788	0.5569	0.3747	0.030*	
C21	0.2049 (5)	0.6011 (3)	0.4174 (3)	0.0157 (11)	
C22	0.2091 (6)	0.6018 (3)	0.3269 (3)	0.0222 (12)	
H22	0.1637	0.6431	0.2935	0.027*	
C23	0.2778 (6)	0.5437 (3)	0.2856 (4)	0.0261 (13)	
H23	0.2804	0.5458	0.2248	0.031*	
C24	0.3423 (6)	0.4830 (3)	0.3338 (4)	0.0251 (12)	
H24	0.3879	0.4427	0.3057	0.030*	
C25	0.3412 (6)	0.4803 (3)	0.4227 (4)	0.0258 (13)	
H25	0.3868	0.4386	0.4554	0.031*	
C26	0.2729 (6)	0.5389 (3)	0.4639 (4)	0.0243 (12)	
H26	0.2725	0.5366	0.5248	0.029*	
C31	0.1333 (6)	0.6589 (3)	0.5800 (3)	0.0197 (11)	
C32	0.0283 (6)	0.6151 (3)	0.6158 (3)	0.0233 (12)	
H32	−0.0569	0.5951	0.5804	0.028*	
C33	0.0503 (7)	0.6009 (3)	0.7051 (4)	0.0274 (13)	
H33	−0.0207	0.5709	0.7303	0.033*	
C34	0.1734 (7)	0.6299 (3)	0.7572 (4)	0.0293 (14)	
H34	0.1883	0.6192	0.8176	0.035*	
C35	0.2751 (7)	0.6748 (3)	0.7201 (4)	0.0318 (14)	
H35	0.3587	0.6961	0.7557	0.038*	
C36	0.2558 (7)	0.6890 (3)	0.6314 (4)	0.0286 (13)	
H36	0.3265	0.7192	0.6063	0.034*	

### Atomic displacement parameters (Å^2^)

**Table:**

	*U*^11^	*U*^22^	*U*^33^	*U*^12^	*U*^13^	*U*^23^
Au	0.02540 (14)	0.01827 (12)	0.02023 (13)	−0.00202 (8)	0.00242 (9)	−0.00004 (8)
Cl1	0.0355 (9)	0.0266 (7)	0.0414 (9)	−0.0078 (6)	0.0081 (7)	0.0040 (6)
P1	0.0232 (8)	0.0201 (7)	0.0192 (7)	0.0002 (6)	0.0027 (6)	0.0008 (5)
S1	0.0387 (9)	0.0204 (7)	0.0198 (7)	−0.0048 (6)	0.0033 (6)	0.0002 (5)
O1	0.025 (2)	0.022 (2)	0.022 (2)	0.0015 (16)	0.0009 (16)	−0.0001 (15)
N1	0.023 (3)	0.021 (2)	0.027 (3)	−0.0022 (19)	0.003 (2)	−0.0017 (19)
C1	0.018 (3)	0.024 (3)	0.020 (3)	−0.002 (2)	0.004 (2)	0.000 (2)
C2	0.031 (3)	0.014 (3)	0.026 (3)	−0.001 (2)	0.004 (2)	−0.003 (2)
C3	0.020 (3)	0.022 (3)	0.025 (3)	0.002 (2)	0.004 (2)	−0.005 (2)
C4	0.026 (3)	0.020 (3)	0.026 (3)	0.000 (2)	0.007 (2)	−0.001 (2)
C5	0.031 (3)	0.020 (3)	0.019 (3)	0.006 (2)	0.005 (2)	0.000 (2)
C6	0.026 (3)	0.026 (3)	0.028 (3)	0.002 (2)	−0.002 (2)	−0.003 (2)
C7	0.024 (3)	0.023 (3)	0.033 (3)	−0.005 (2)	0.006 (3)	0.000 (2)
C8	0.033 (4)	0.030 (3)	0.023 (3)	0.001 (3)	−0.001 (3)	−0.003 (2)
C11	0.023 (3)	0.022 (3)	0.016 (3)	−0.002 (2)	0.001 (2)	0.002 (2)
C12	0.029 (3)	0.023 (3)	0.025 (3)	0.002 (2)	0.006 (3)	−0.002 (2)
C13	0.030 (4)	0.034 (3)	0.032 (3)	0.009 (3)	0.005 (3)	0.000 (3)
C14	0.019 (3)	0.046 (4)	0.023 (3)	−0.006 (3)	−0.004 (2)	0.004 (3)
C15	0.029 (3)	0.033 (3)	0.023 (3)	−0.005 (3)	0.000 (2)	−0.003 (2)
C16	0.024 (3)	0.024 (3)	0.026 (3)	0.001 (2)	0.000 (2)	0.004 (2)
C21	0.007 (3)	0.019 (3)	0.019 (3)	−0.009 (2)	−0.005 (2)	0.006 (2)
C22	0.022 (3)	0.021 (3)	0.023 (3)	−0.003 (2)	0.001 (2)	−0.003 (2)
C23	0.028 (3)	0.031 (3)	0.020 (3)	−0.005 (3)	0.004 (2)	−0.002 (2)
C24	0.025 (3)	0.018 (3)	0.032 (3)	0.002 (2)	0.006 (2)	−0.006 (2)
C25	0.027 (3)	0.019 (3)	0.030 (3)	0.002 (2)	0.001 (3)	0.007 (2)
C26	0.027 (3)	0.024 (3)	0.022 (3)	−0.001 (2)	0.002 (2)	−0.002 (2)
C31	0.020 (3)	0.016 (3)	0.023 (3)	−0.002 (2)	0.005 (2)	−0.005 (2)
C32	0.027 (3)	0.015 (3)	0.026 (3)	−0.002 (2)	−0.003 (2)	0.000 (2)
C33	0.031 (3)	0.026 (3)	0.026 (3)	−0.001 (3)	0.005 (3)	0.002 (2)
C34	0.039 (4)	0.026 (3)	0.021 (3)	0.001 (3)	0.000 (3)	0.003 (2)
C35	0.030 (4)	0.040 (3)	0.024 (3)	−0.013 (3)	−0.004 (3)	−0.002 (3)
C36	0.031 (4)	0.033 (3)	0.023 (3)	−0.004 (3)	0.006 (3)	0.001 (2)

### Geometric parameters (Å, º)

**Table:**

Au—P1	2.2535 (14)	C13—H13	0.9500
Au—S1	2.3070 (14)	C14—C15	1.372 (8)
Cl1—C4	1.756 (6)	C14—H14	0.9500
P1—C21	1.783 (5)	C15—C16	1.378 (8)
P1—C11	1.811 (6)	C15—H15	0.9500
P1—C31	1.824 (5)	C16—H16	0.9500
S1—C1	1.764 (5)	C21—C26	1.402 (7)
O1—C1	1.362 (6)	C21—C22	1.413 (7)
O1—C8	1.443 (6)	C22—C23	1.390 (7)
N1—C1	1.274 (6)	C22—H22	0.9500
N1—C2	1.418 (7)	C23—C24	1.383 (7)
C2—C3	1.389 (8)	C23—H23	0.9500
C2—C7	1.402 (8)	C24—C25	1.386 (8)
C3—C4	1.387 (7)	C24—H24	0.9500
C3—H3	0.9500	C25—C26	1.394 (7)
C4—C5	1.378 (8)	C25—H25	0.9500
C5—C6	1.395 (8)	C26—H26	0.9500
C5—H5	0.9500	C31—C36	1.379 (7)
C6—C7	1.398 (7)	C31—C32	1.389 (7)
C6—H6	0.9500	C32—C33	1.399 (7)
C7—H7	0.9500	C32—H32	0.9500
C8—H8A	0.9800	C33—C34	1.380 (8)
C8—H8B	0.9800	C33—H33	0.9500
C8—H8C	0.9800	C34—C35	1.389 (8)
C11—C12	1.398 (8)	C34—H34	0.9500
C11—C16	1.409 (7)	C35—C36	1.391 (8)
C12—C13	1.383 (8)	C35—H35	0.9500
C12—H12	0.9500	C36—H36	0.9500
C13—C14	1.391 (8)		
			
P1—Au—S1	175.62 (5)	C15—C14—C13	120.2 (6)
C21—P1—C11	105.5 (2)	C15—C14—H14	119.9
C21—P1—C31	105.8 (2)	C13—C14—H14	119.9
C11—P1—C31	106.2 (2)	C14—C15—C16	120.8 (5)
C21—P1—Au	114.80 (17)	C14—C15—H15	119.6
C11—P1—Au	111.17 (17)	C16—C15—H15	119.6
C31—P1—Au	112.75 (17)	C15—C16—C11	119.8 (5)
C1—S1—Au	101.78 (18)	C15—C16—H16	120.1
C1—O1—C8	115.4 (4)	C11—C16—H16	120.1
C1—N1—C2	120.8 (5)	C26—C21—C22	117.0 (5)
N1—C1—O1	119.7 (5)	C26—C21—P1	124.8 (4)
N1—C1—S1	127.7 (4)	C22—C21—P1	118.2 (4)
O1—C1—S1	112.6 (4)	C23—C22—C21	121.8 (5)
C3—C2—C7	119.6 (5)	C23—C22—H22	119.1
C3—C2—N1	119.9 (5)	C21—C22—H22	119.1
C7—C2—N1	120.1 (5)	C24—C23—C22	119.4 (5)
C4—C3—C2	118.8 (5)	C24—C23—H23	120.3
C4—C3—H3	120.6	C22—C23—H23	120.3
C2—C3—H3	120.6	C23—C24—C25	120.6 (5)
C5—C4—C3	123.2 (5)	C23—C24—H24	119.7
C5—C4—Cl1	118.5 (4)	C25—C24—H24	119.7
C3—C4—Cl1	118.2 (4)	C24—C25—C26	119.8 (5)
C4—C5—C6	117.6 (5)	C24—C25—H25	120.1
C4—C5—H5	121.2	C26—C25—H25	120.1
C6—C5—H5	121.2	C25—C26—C21	121.5 (5)
C5—C6—C7	120.8 (5)	C25—C26—H26	119.3
C5—C6—H6	119.6	C21—C26—H26	119.3
C7—C6—H6	119.6	C36—C31—C32	120.8 (5)
C6—C7—C2	119.9 (5)	C36—C31—P1	118.4 (4)
C6—C7—H7	120.0	C32—C31—P1	120.7 (4)
C2—C7—H7	120.0	C31—C32—C33	118.8 (5)
O1—C8—H8A	109.5	C31—C32—H32	120.6
O1—C8—H8B	109.5	C33—C32—H32	120.6
H8A—C8—H8B	109.5	C34—C33—C32	121.0 (5)
O1—C8—H8C	109.5	C34—C33—H33	119.5
H8A—C8—H8C	109.5	C32—C33—H33	119.5
H8B—C8—H8C	109.5	C33—C34—C35	119.1 (5)
C12—C11—C16	119.0 (5)	C33—C34—H34	120.5
C12—C11—P1	118.1 (4)	C35—C34—H34	120.5
C16—C11—P1	122.9 (4)	C34—C35—C36	120.7 (5)
C13—C12—C11	120.2 (5)	C34—C35—H35	119.7
C13—C12—H12	119.9	C36—C35—H35	119.7
C11—C12—H12	119.9	C31—C36—C35	119.5 (5)
C12—C13—C14	120.0 (6)	C31—C36—H36	120.2
C12—C13—H13	120.0	C35—C36—H36	120.2
C14—C13—H13	120.0		
			
C2—N1—C1—O1	−175.5 (5)	C12—C11—C16—C15	0.8 (8)
C2—N1—C1—S1	6.8 (8)	P1—C11—C16—C15	179.0 (4)
C8—O1—C1—N1	−2.1 (7)	C11—P1—C21—C26	−110.0 (5)
C8—O1—C1—S1	176.0 (4)	C31—P1—C21—C26	2.3 (5)
Au—S1—C1—N1	−153.4 (5)	Au—P1—C21—C26	127.3 (4)
Au—S1—C1—O1	28.7 (4)	C11—P1—C21—C22	69.0 (4)
C1—N1—C2—C3	−113.5 (6)	C31—P1—C21—C22	−178.7 (4)
C1—N1—C2—C7	73.4 (7)	Au—P1—C21—C22	−53.7 (4)
C7—C2—C3—C4	−1.4 (8)	C26—C21—C22—C23	−0.2 (7)
N1—C2—C3—C4	−174.6 (5)	P1—C21—C22—C23	−179.3 (4)
C2—C3—C4—C5	0.3 (8)	C21—C22—C23—C24	0.8 (8)
C2—C3—C4—Cl1	−180.0 (4)	C22—C23—C24—C25	−1.1 (8)
C3—C4—C5—C6	0.7 (8)	C23—C24—C25—C26	0.7 (8)
Cl1—C4—C5—C6	−179.0 (4)	C24—C25—C26—C21	−0.1 (8)
C4—C5—C6—C7	−0.6 (8)	C22—C21—C26—C25	−0.2 (8)
C5—C6—C7—C2	−0.6 (8)	P1—C21—C26—C25	178.8 (4)
C3—C2—C7—C6	1.6 (8)	C21—P1—C31—C36	90.7 (5)
N1—C2—C7—C6	174.7 (5)	C11—P1—C31—C36	−157.5 (4)
C21—P1—C11—C12	−169.0 (4)	Au—P1—C31—C36	−35.5 (5)
C31—P1—C11—C12	79.0 (5)	C21—P1—C31—C32	−89.3 (5)
Au—P1—C11—C12	−44.0 (5)	C11—P1—C31—C32	22.4 (5)
C21—P1—C11—C16	12.7 (5)	Au—P1—C31—C32	144.4 (4)
C31—P1—C11—C16	−99.3 (5)	C36—C31—C32—C33	−1.1 (8)
Au—P1—C11—C16	137.7 (4)	P1—C31—C32—C33	179.0 (4)
C16—C11—C12—C13	0.6 (8)	C31—C32—C33—C34	0.2 (8)
P1—C11—C12—C13	−177.7 (4)	C32—C33—C34—C35	1.1 (9)
C11—C12—C13—C14	−0.8 (9)	C33—C34—C35—C36	−1.7 (9)
C12—C13—C14—C15	−0.3 (9)	C32—C31—C36—C35	0.6 (9)
C13—C14—C15—C16	1.7 (9)	P1—C31—C36—C35	−179.5 (5)
C14—C15—C16—C11	−1.9 (8)	C34—C35—C36—C31	0.8 (9)

### Hydrogen-bond geometry (Å, º)

Hydrogen-bond geometry (Å, °) for (I), Form β, *Cg*1, *Cg*3 and *Cg*4 are the centroids of the C2–C7, C21–C26 and C31–C36 rings, respectively.

**Table:**

*D*—H···*A*	*D*—H	H···*A*	*D*···*A*	*D*—H···*A*
C3—H3···O1^i^	0.95	2.47	3.315 (7)	148
C5—H5···*Cg*4^ii^	0.95	2.85	3.492 (6)	126
C12—H12···*Cg*1^ii^	0.95	2.64	3.450 (6)	143
C14—H14···*Cg*3^iii^	0.95	2.80	3.570 (6)	139
C23—H23···*Cg*1^iv^	0.95	2.65	3.435 (6)	140

Symmetry codes: (i) −*x*+1, −*y*+2, −*z*+1; (ii) −*x*, −*y*+2, −*z*+1; (iii) *x*−1, *y*, *z*; (iv) −*x*+1/2, *y*−1/2, −*z*+1/2.
